# Expansion of a highly sensitive and specific ELISA test for diagnosis of hydatidosis using recombinant EgB8/2 protein

**DOI:** 10.22038/ijbms.2018.29024.7021

**Published:** 2019-02

**Authors:** Sareh Bashiri, Fahimeh Nemati Mansoor, Zarrintaj Valadkhani

**Affiliations:** 1 Department of Biotechnology, Faculty of Advanced Sciences and Technology, Tehran Medical Sciences, Islamic Azad University, Tehran, Iran; 2 Department of Parasitology, Pasteur Institute of Iran, Tehran, Iran

**Keywords:** Diagnosis, Echinococcus, ranulosus ELISA, Hydatidosis, Recombinant EgB8/2 – protein

## Abstract

**Objective(s):**

Hydatidosis is a zoonotic infection and endemic in Iran. Due to the serological cross-reactivity (of sera) with other parasitic infection, diagnosis of hydatid cyst is considered to be problematic. In this regard, application of recombinant antigens improves serological diagnosis for human hydatidosis. Here, we present an ELISA test based on B8/2 recombinant antigen of *Echinococcus granulosus* with particular regard to its capability to diagnose human hydatidosis.

**Materials and Methods:**

The synthesized *E. granulosus *B8/2 (EgB8/2) gene was sub-cloned into pET28b (+) plasmid. Nde1 and Hind3 restriction enzymes were used to confirm the recombinant plasmid extraction. Cloning was verified by colony PCR, digestion enzymes, and sequence determination methods. To express rtEgB8/2, strains of *Escherichia coli *BL21 (DE3) pLysS and Rosetta (DE3) were induced with isopropyl β-D-1-thiogalactopyranoside (IPTG). A Ni-NTA column was used for purification, and the expressed protein was analyzed by SDS-PAGE as well as western blotting. ELISA test was used to identify the antigenicity of produced protein.

**Results:**

The presence of EgB8/2 gene fragment in the recombinant plasmid was confirmed. SDS-PAGE showed that the BL21 (DE3) pLysS strain had the highest level of expression and a protein band of 11 kDa was observed in induced bacteria. Western blotting approved the purity of rtEgB8/2 protein, and ELISA test measured sensitivity and specificity as 95% and 97.5%, respectively.

**Conclusion:**

*E. granulosus* metacestode contains a high amount of antigen B protein. These results confirm the reproducibility of high-quality rtEgB8/2 recombinant antigen as a reliable candidate in serological test.

## Introduction

*Echinococcus granulosus* is responsible for human hydatidosis and it is considered a major concern in several regions of the world including Iran (1-4). Humans are accidentally infected by drinking water, consumption of vegetables and foods contaminated with the tapeworm’s eggs, or even by licking and touching infected dogs. Although all tissues and organs may be affected via the blood and the lymphatic systems, expansion of the parasite’s metacestode mostly happens in the liver and the lungs (5). As the infection may remain asymptomatic for a remarkably long period of time, several methods such as imaging (ultrasonography or radiology), physical examinations, and serological tests are applied for the primary diagnosis of hydatidosis (3, 4, 6).

Several kinds of antigens such as antigen B (AgB), antigen 5, and hydatid cyst fluid have been used for the diagnosis of hydatidosis, but their efficiency is not sufficient. AgB is remarkably found in the hydatid cystic fluid and is a highly immunogenic lipoprotein (2, 3, 7-13). ELISA, PCR, and western blotting tests are widely applied to diagnose the disease; however, due to false negative results in PCR this method is not widely used (9). The serological tests of hydatidosis are influenced by the cross-reactivity between *E. granulosus* and other parasitic infections such as *Echinococcus multilocularis*, *Taenia solium, and Fasciola hepatica*, which result in reduced performance that is why recombinant antigen-based diagnostic methods are recently sought (10).

Recombinant antigens represent a higher diagnostic odds ratio compared to other evaluated antigens, thus they have been suggested for diagnosis of infection in several hydatidosis-endemic areas (4). Previous studies have shown that among the antigens that are used to detect hydatidosis, the EgB8/2 antigen, in the fluid of the hydatid cysts, is very immunogenic. Between the 5 subunits of this antigen, the 8 kDa subunit provides the best diagnostic specificity and sensitivity with the patient’s sera (10, 14). Due to the signal peptides and a cleavage signal from eukaryotic peptidase, the EgB8/2 sequence shows a hydrophobic region from position 1 – 20. This indicates that the polypeptide (EgB8/2) can be a secreted protein by the scolex in the hydatid cyst (15). Since recombinant proteins with signal peptide region at the N-terminal can result in decreasing of recombinant protein expression and inclusion body formation in *Escherichia coli *system (16), elimination of this region in EgB8/2 protein leads to enhancing the yield of soluble recombinant protein expression.

Due to the improvements in methods applied for the production of synthetic recombinant antigens, i.e. cloning, expression and purification, these antigens have shown a good performance and might be less susceptible to interact with other parasitic antibodies. In this study, an ELISA test was developed to diagnose certain antibodies in the infected patient’s serum samples using rtEgB8/2 protein with the highest antigenicity.

## Materials and Methods

***Strains of bacteria and plasmid***

For cloning and expression of the recombinant tEgB8/2 protein, *Escherichia coli* DH5α strain (Invitrogen, Carlsbad, CA, USA), BL21(DE3) pLysS, and Rosetta (DE3) (Promega, Madison, WI, USA) were applied.

***Recombinant expression*** ***plasmid construction*** ***and*** ***sequence analysis***

The sequence for the EgB8/2 gene was obtained from the GenBank site (accession number: EGU15001) and a truncated fragment of the gene (222 bp), wherein the hydrophobic amino acids in signal peptide have been removed, (21–90) was designed. Meanwhile in order to facilitate the cloning procedure, sites of the cleavage enzymes (HindIII and NdeI) were determined at the end of 3’ and 5’ genes, respectively, and sent for recombinant vector synthesis (Biomatic co., Canada). The truncated fragment was digested using HindIII and NdeI enzymes and a pET-28b (+) expression vector was used for sub-cloning. To confirm the insertion of tEgB8/2 gene in the recombinant plasmid, several methods such as colony-PCR, enzymatic digestion, and finally a sequence determination (Macrogen co., Seoul, Korea) was conducted. pET-28b (+)-EgB8/2 is used to refer to the recombinant plasmid, which contains the truncated sequence of EgB8/2.

***Expression of recombinant EgB8/2 (rtEgB8/2)***

Heat shock was used to transform pET-28b (+)-EgB8/2 into *E. coli *Rosetta (DE3) and BL21 (DE3) pLysS strains. Luria Bertani (LB) broth with 35 μg/ml chloramphenicol and 25 μg/ml kanamycin was used for bacterial growth (30 ^°^C, shaking at 160 rpm). We used different concentrations of isopropyl-β-D-thiogalactoside (IPTG) (from 0.2 to 1 mM) at optical density 600 nm (OD_600_) of 0.4–1 to induce the expression of rtEgB8/2. To detect the optimal expression situation according to the 12% SDS-PAGE analysis, bacteria were incubated for different times (i.e. 2, 4, 8, and 16 hr) by vigorous shaking.

***Purification of rtEgB8/2 by immobilized metal affinity chromatography (IMAC)***

Bacteria in 500 ml culture media were centrifuged, and after discarding the supernatant, the cells were resuspended in 20 ml of binding buffer (0.5 M NaCl, 30 mM Tris-base, Urea 8 M, and Imidazole 2 mM, pH=8). In addition, 1mM Phenylmethylsulfonyl Fluoride (PMSF) was used to inhibit the protease activity. Cells were sonicated for 12-13 min on ice, shook for 2 hr, and then centrifuged (6848 ×g, 20 min, 4 ^°^C). A 5 ml column His-Trap™ FF (GE) was used to purify the supernatant by Ni+2-NTA affinity chromatography. After that, 5-10 fold water was added for each volume of resin. Resin was washed with 40 ml binding buffer. Then, the supernatant of rtEgB8/2 protein was added and washed with washing buffers W_1 _(20 mM imidazole), W_2 _(40 mM imidazole), and W_3_ (60 mM imidazole). Finally, E_1 _and E_2 _elution buffers (binding buffer containing 300 mM imidazole, 1 M urea and 500 mM imidazole, 0.5 M urea) were applied for eluting the rtEgB8/2 protein. 

***Serum samples***

In this study, the application of rtEgB8/2 as a serological antigen identification probe was investigated on 40 conﬁrmed chronic hydatidosis positive serum samples and 40 negative controls from healthy individuals. Moreover, 40 cases of fasciolosis, toxocariasis, amebiasis, and toxoplasmosis were also used to investigate the possible cross-reactivity during the process.

***Western blot analysis***

After loading the prepared samples into the 12% SDS-PAGE wells and performing the electrophoresis, the running was stopped when most signs of the protein marker were observed on the gel. For analyzing the results, PVDF membranes (Millipore, Billerica, MA, USA) were used for protein band transferring. A 2% bovine serum albumin (BSA) in phosphate-buffered saline (PBS; 10X) was used to block the membrane. Following Tween 20 treatments, proteins were probed with 1:50 diluted pooled human sera, descriptive of chronic and non-infected hydatid disease. Anti-human IgG antibodies conjugated to horseradish peroxidase (HRP) with a 1:10,000 dilution was used for antibody detection. A blocking buffer was used to dilute the human serum samples and the secondary antibodies, and then diaminobenzoic acid (DAB) (Kem-En-Tec, Taastrup, Denmark) and hydrogen peroxide (H_2_O_2_) were used for observations.

***ELISA***

A 50 *µl* of rtEgB8/2 antigen in 10 mM coating buffer (pH=9.6) was used to coat the Maxisorp microtiter ELISA plates (Maxi- sorp, Nunc, Roskilde, Denmark). Plates were then stored at 4*°C* overnight. Plates were washed twice with washing buffer (each time 300 *µl)*, and were then blocked with PBS (10X) containing 1% BSA with Tween 20 at room temperature for 2 hr to prevent non-specific bindings. Then, the procedure was continued as mentioned previously (15).

***Statistical analysis***

GraphPad.prism.v7.01 software was used for statistical analysis. To calculate the sensitivity, the number of true positive was added to the number of false negative, and the specificity was expressed as the number of true negative / number of true negative + number of false positive. The intensity of each protein band was measured using NIH ImageJ analysis software (17).

## Results

***Gene fragment cloning***

**Figure 1:**
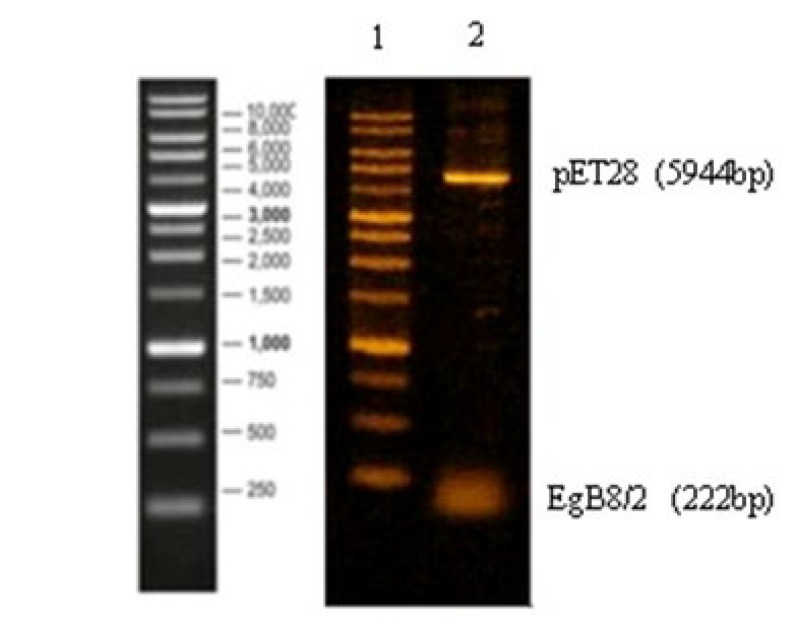
Verification of recombinant plasmid pET28b (+)-rtEgB8/2 by enzymatic digestion. Lane1: 1 kb DNA size marker, lane 2: Nde1/HindIII digested pET28b (+)-rtEgB8/2

A 222 bp gene fragment of tEgB8/2 encoding amino acids 21 to 90 (accession no. EGU15001) from the genome of *E. granulosus* was used in this study. Colony-PCR and restriction enzymes methods were used to confirm the recombinant clone, which were sub-cloned into pET-28b (+) expression plasmid (Figure 1). The sequence analysis showed that rtEgB8/2 gene was identical to the sequence provided in the GenBank database.

**Figure 2:**
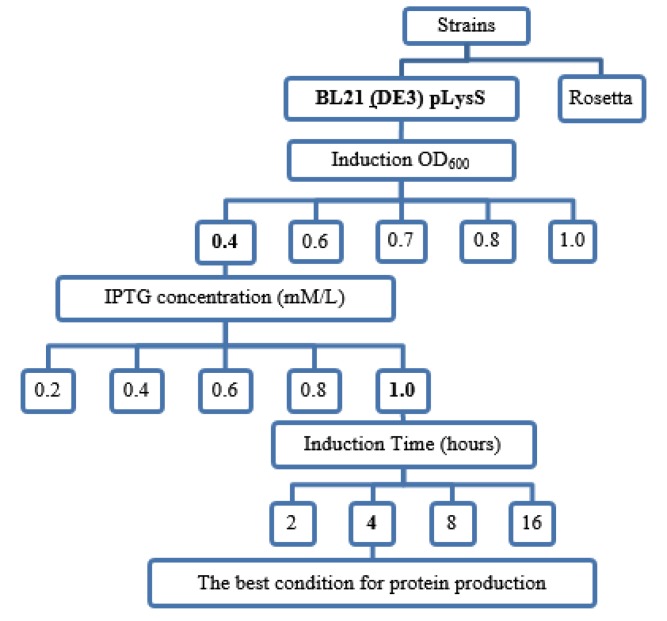
Schematic diagram shows the experimental optimization process. The rtEgB8/2 gene was expressed at different concentrations of isopropyl β-D-1-thiogalactopyranoside (IPTG), induction optical density (OD), and incubation time. IPTG at the concentrations from 0.2-1 mM was used to induce BL21 (DE3) pLysS bacteria strain. The incubation time varied between 2, 4, 8, and 16 hr

**Figure 3:**
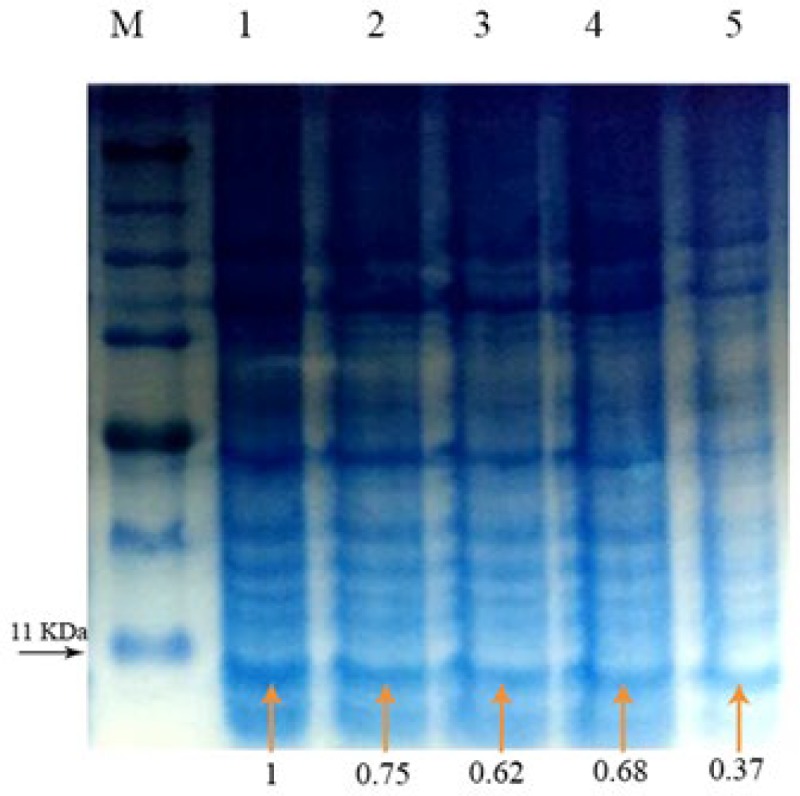
SDS-PAGE showing the expression of rtEgB8/2 gene at varied isopropyl β-D-1-thiogalactopyranoside (IPTG) concentrations. IPTG at concentrations from 0.2 mM (lane5) to 1 mM (lane 1) were used to induce BL21 (DE3) pLysS bacteria. The intensity of each protein band (orange arrows) was quantified by densitometry using ImageJ analysis software. Induction with 1 mM IPTG showed the highest expression in induced *Escherichia coli *Bl21 (DE3) pLysS strain (lane1)

**Figure 4:**
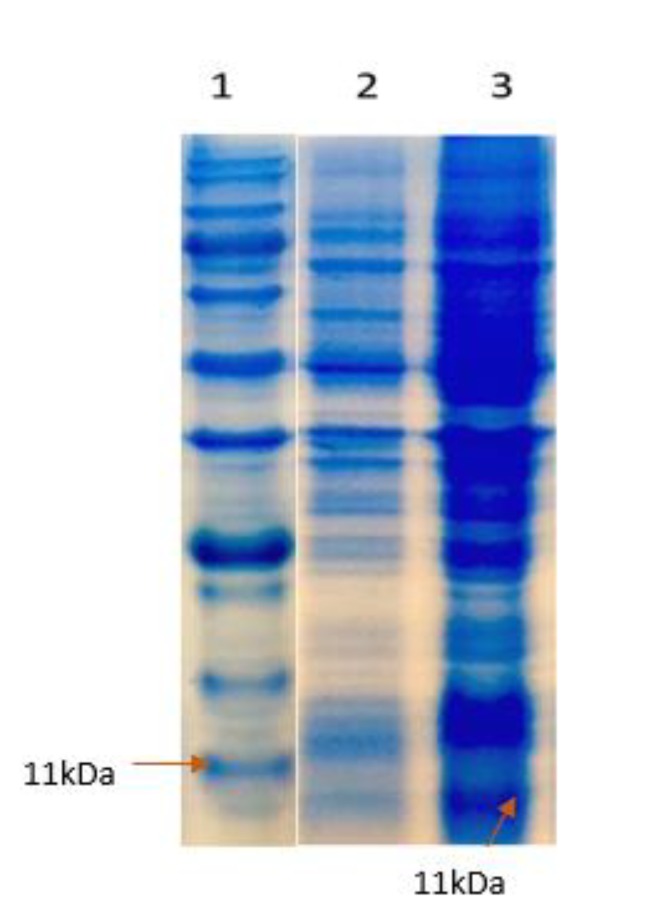
The expressed protein in *Escherichia coli* BL21 (DE3) pLysS is visualized by coomassie blue-stained SDS-PAGE. 1: Marker 2: Before induction, 3: After induction with isopropyl β-D-1-thiogalactopyranoside (IPTG)

**Figure 5:**
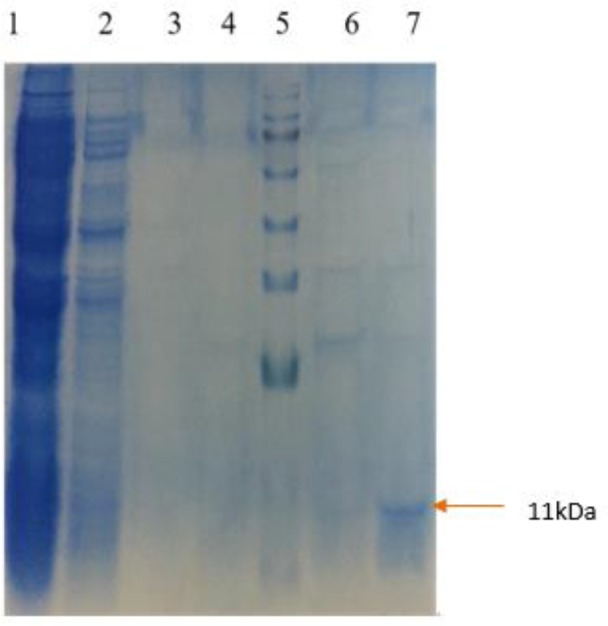
Purification of rtEgB8/2 was applied using a Ni+2-NTA affinity chromatography. The recombinant protein (rtEgB8/2) was analyzed using SDS-PAGE. Lane1: flow through; lane 2: Wash 1; lane 3: Wash 2; lane 4: Wash 3; lane 5: Marker; lane 6: Elution 1; lane 7: Elution 2

**Figure 6:**
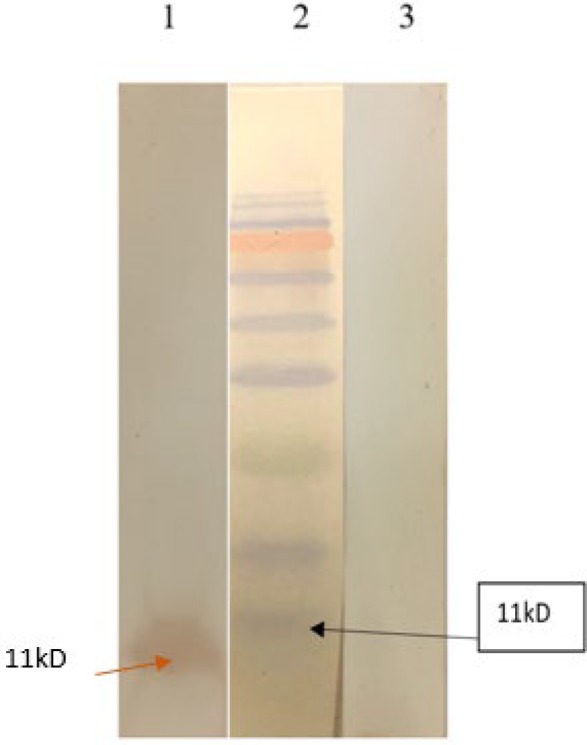
Immunogenicity evaluation of rtEgB8/2. A 12% SDS-PAGE analysis was performed on purified rtEgB8/2 proteins. Protein bands were transferred onto PVDF membranes and pooled sera from human hydatidosis was used for probing. Marker (lane 1), positive serum (lane 2), and non-*Echinococcus granulosus* infection serum (lane 3)

**Figure 7:**
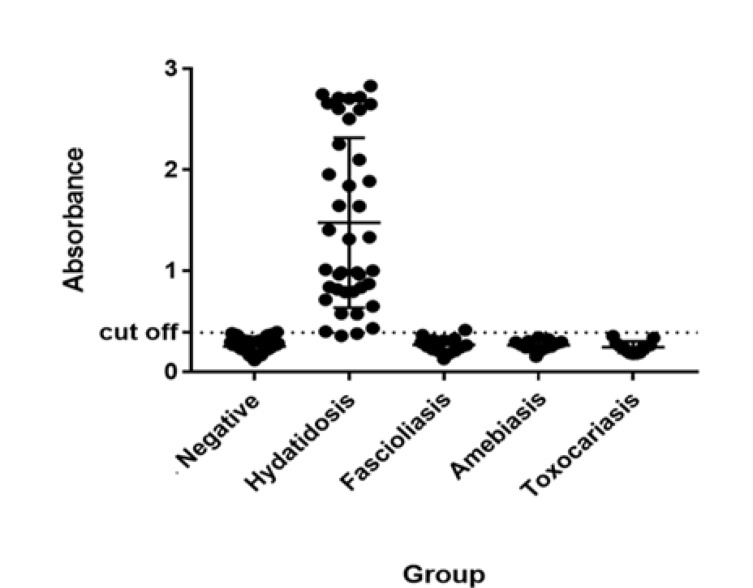
The comparison of ELISA reactivity with rtEgB8/2 antigen using scatter of rtEgB8/2 ELISA absorbance value with hydatidosis, negative sera, and other parasitic diseases . The cut-off value for rtEgB8/2 is 0.392, which was indicated by the dashed lines

**Table 1:** Expression of rtEgB8/2 gene in Escherichia coli BL21 (DE3) pLysS and Rosetta (DE3) as two different bacterial expression hosts

Strains	Optical Density (OD_600_)	IPTG (mM/lit.)	Time (hr)	Yield (%)
Bl21 (DE3) pLysS	0.4	0.2	4.0	1.0
Rosetta (DE3)	0.4	0.2	4.0	0.28

IPTG: Isopropyl β-D-1-thiogalactopyranoside.

***Optimized expression of rtEgB8/2***

Results showed that *E. coli* BL21 (DE3) pLysS strain had the highest level of protein expression, so we decided to use this strain to continue the experiment. After transfection, the grown bacteria in LB broth media were induced with 1 mM of IPTG. In order to obtain optimized expression of rtEgB8/2 protein, the one-factor-at-a-time (OFAT) method was applied. Recombinant plasmid’s gene expression levels were investigated in several conditions including different strains of *E. coli*, various concentrations of IPTG, and different incubation times. The highest expression level was observed in the induction of* E. coli* Bl21 (DE3) pLysS strain with a regime of: 1 mM IPTG, an OD of 0.4 at 600 nm (OD_600_), and a 4 hr duration time maintained at 30 ^°^C (Table 1, Figure 2). By analyzing the SDS-PAGE, results showed a protein band at 11 kDa in the induced bacteria. The intensity of each protein band was then calculated. The relative intensity of each protein band was measured as a ratio of each protein band to the lane^,^s loading control. 

SDS- PAGE was used to evaluate the induced bacteria. The protein intensity (%) was assessed using ImageJ analysis software. The quantity of each protein band reflects the relative amounts as a ratio. Induced *E. coli* Bl21 (DE3) plysS strain showed the highest expression.

The intensity of each protein band was quantified by densitometry using ImageJ analysis software (Figure 3). The highest expression was observed in 1 mM IPTG induction of *E. coli* BL21 (DE3) pLysS strain.

***Purification of recombinant rtEgB8/2 protein***

Sonication in 500 ml culture media was used to harvest the induced bacteria. To purify the recombinant protein (rtEgB8/2), an IMAC on a Ni²+-NTA column was applied, and the proteins were analyzed by SDS-PAGE (Figure 4 and 5).

***Immunoblotting***

Immunoblotting probed with pooled human sera of hydatidosis individuals was used to confirm the antigenicity of the recombinant protein (Figure 6).

***ELISA***

ELISA test was employed to assess the sensitivity and specificity of the purified recombinant antigen (rtEgB8/2) to sera from hydatidosis patients, non-hydatidosis, and healthy individuals. The results from the ELISA test revealed a mean OD and standard deviation (SD) of the negative sera as 0.254 and 0.069, respectively. To distinguish between the positive and negative reactions, the cut-off (mean+2SD) point was also calculated as 0.392. To measure the sensitivity and the specificity of the ELISA test, the formulas as mentioned in the statistical analysis section were used. Among 40 positive sera, there were two false negative reactions with rtEgB8/2 antigen, thus the sensitivity of this protein for diagnosis of hydatidosis was 95%. Out of 40 other parasitic disease patients, only one serum from a fascioliasis patient showed false positive, so the specificity was calculated as 97.5%. The results of all 40 negative sera were about or under cut-off value (Figure 7). This statistical analysis confirmed that the rtEgB8/2 protein has a better performance compared to other recombinant antigens in the ELISA test. 

## Discussion

In recent century, a few immunodiagnostic tests have been developed. Ag5 and AgB are among the most common antigens used to detect human hydatidosis; however, their inefficient performance in diagnosis of human hydatidosis is considered a critical problem.

In the present study, a truncated EgB8/2 gene from *E. granulosus* was expressed in various bacteria strains, and after purification it was used as a recombinant antigen for the diagnosis of hydatidosis. Western blot and ELISA tests were carried out to investigate the antigenicity of the protein.

In recent years, different genes have been cloned and expressed from various stages of *E.* *granulosus* by several research groups and with various aims that include designing adequate DNA vaccine or recombinant antigens for serological diagnosis (14). We have chosen the smallest subunit (8 kDa) as the best diagnosis antigen in hydatidosis. In most studies, the results indicated that antigens from the hydatid fluid are more sensitive than other antigens (3, 4).

Results showed that the highest level of rtEgB8/2 protein expression was in culture media with 1 mM concentration of IPTG, 4 hr after induction at OD_600_=0.4 with a temperature of 30°C. The attendance of 6 Histidine tags allowed the purification of rtEgB8/2 protein via IMAC on the Ni²+-NTA column at the optimized pH. The straightforward recombinant protein purification using IMAC results in a very pure protein as previously mentioned (18). SDS-PAGE was used to analyze the eluted fractions and the results revealed a protein band of about 11 kDa, which is in an agreement with the estimated molecular mass of rtEgB8/2 protein. 

The antigenicity and the immunoreactivity of the purified rtEgB8/2 were confirmed using the sera of the patients with hydatidosis by western blotting and ELISA technique.

Through previous results, we found out that many non-specific components are included in the native antigens’ structure that interact with other parasitic or even non-parasitic infections (19-26), which is why we have used recombinant antigens. Kalantari *et al.* reported that the antigenic activity of recombinant AgB in ELISA test was positive with 91% (33/36) of the sera from the patients with hydatidosis (3). An anti-IgG ELISA was performed by Rott *et al*. to compare the sensitivity of native AgB to rAgB8/1 and rAgB8/2 recombinant antigens (15). They found that the native AgB holds a sensitivity of 77.41% and a specificity of 81.9%, while rAgB8/1 showed a sensitivity rate of 54.84% and specificity of 80.17%. Also, 83.87% sensitivity and 98.28% specificity was observed for rAg8/2. Furthermore, the results the superior performance of rAgB8/2 over rAgB8/1 and native AgB in ELISA test. Although their sensitivity is slightly higher than our results, but the specificity of our antigen is more significant than the report of Rott *et al* (27). 

Mcvie *et al.* reported 65% sensitivity by using rtEgB antigen in ELISA system from patients with hydatidosis (28). Despite these differences, *E. granulosus* is reported to have an EgB subunit (8 kDa) as its specific antigen with a certain degree of cross-reactivity (27, 29). 

By considering the previous results, our synthesized recombinant tEgB8/2 protein seems to be the most sensitive and speciﬁc antigen for detection of hydatidosis, and its usefulness was evaluated by serodiagnosis antigen using ELISA system (3, 9, 30, 31). Among the 40 positive serum samples used in this study, only two cases in the whole IgG ELISA tests did not react with the recombinant tEgB8/2, meanwhile non-hydatidosis sera were also used to calculate the specificity value. 

In order to improve the accuracy of diagnostic systems, standard methods and specific antigens should be developed (32). To do so, this experiment proposes a versatile method for hydatid diagnosis using rtEgB8/2 protein.

## Conclusion

The efficiency of the current immunodiagnostic antigens in detection of hydatidosis is not adequate; therefore, finding an efficient disease diagnosis methods based on serological tests was the topic of this study. Diagnostic serological tests are progressing in terms of performance as new antigens are introduced and assessed. Consequently, in this study we report the production and the purification of rtEgB8/2 from *E. granulosus,* which was expressed in *E. coli *BL21 (DE3) pLysS strain, using IMAC on Ni²+-NTA sepharose beads. The obtained pure protein can be utilized to develop ELISA tests for diagnostic purpose. 

An interesting new perspective on development of serological tests for detecting human hydatidosis is the use of recombinant or synthetic proteins alone or in combination with other *E. granulosus* antigen to improve the accuracy of diagnostic tests. 
